# Multistability in large scale models of brain activity

**DOI:** 10.1186/1471-2202-14-S1-P84

**Published:** 2013-07-08

**Authors:** Mathieu Golos, Viktor K Jirsa, Emmanuel Daucé

**Affiliations:** 1 Inserm, Aix-Marseille Université, INS UMR 1106, Marseille 13005, France; 2 Ecole Centrale Marseille, Marseille 13013, France

## 

Rich spatiotemporal dynamical patterns, observed in the brain at rest, reveal several large-scale functional networks, presumably involved in different brain functions. In parallel, structural networks obtained by Diffusion Spectrum Imaging ("connectome") identify several interconnected sub-networks that overlap with the functional networks [3]. Neural mass model simulations aim at developing realistic models of the brain activity. In particular, multiple fixed-point attractors can be identified and spontaneous alternation between several brain "states" can be obtained [2]. The number and the organization of the "fixed-point" attractors strongly vary depending of the parameters used in the model.

**Figure 1:**
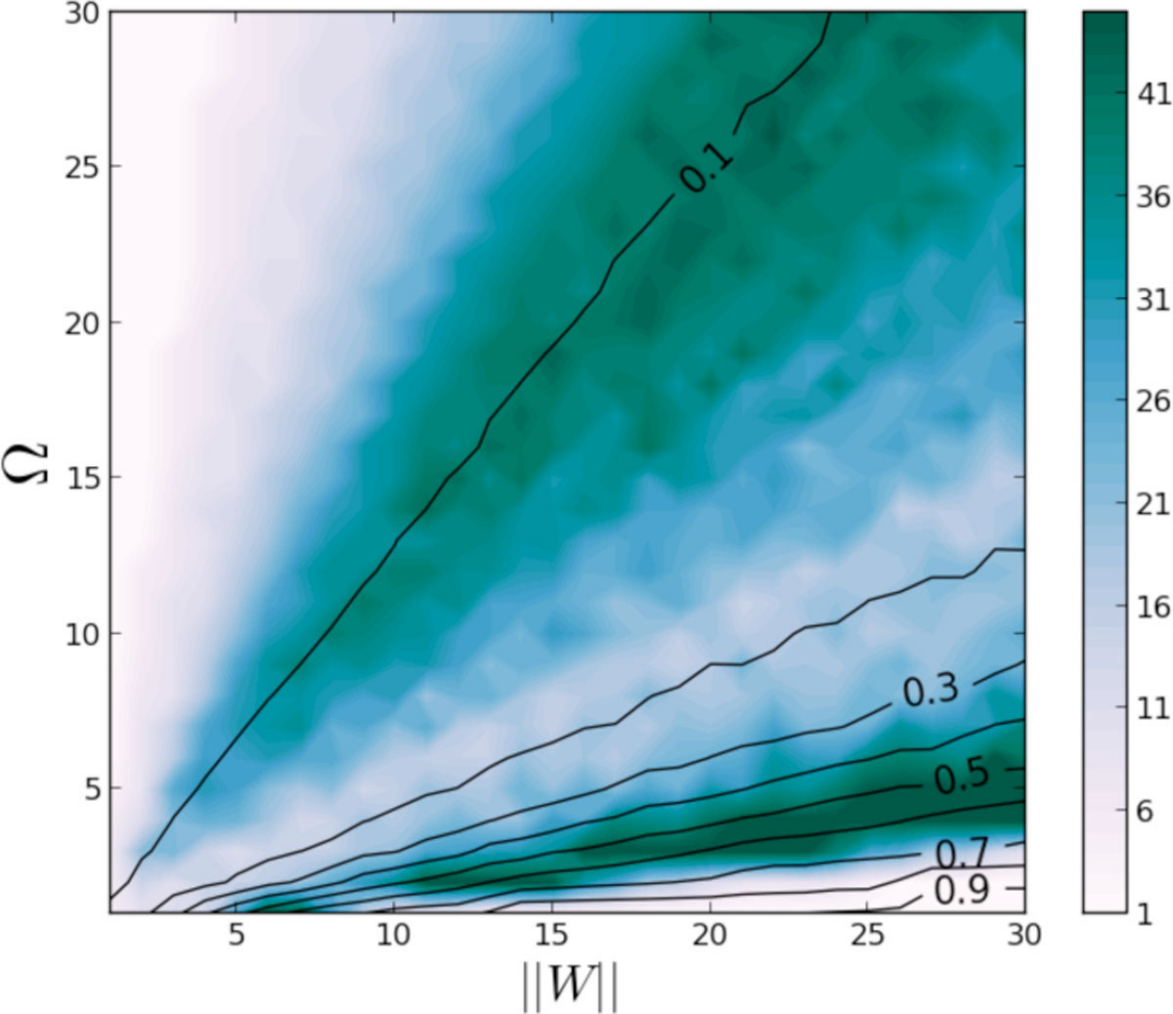
**Number of attractors obtained for different (Ω, ||W||) couples with γ = 30**. Attractor patterns isodensity lines are indicated in black

In order to provide a general view of connectome-based dynamical systems, we developed a simplified neural mass model inspired by the continuous Hopfield network [4], to which we add a stochastic component, and a dynamic threshold (eq.2) that prevents reaching the two trivial attractors of system (eq.1).

(1) $$\tau \frac{dx_{i}}{dt}=-x_{i}+\overset{N}{\underset{j=1}{\sum }}W_{i,j}\varphi \left(x_{j},\gamma ,\theta \right)+\sigma^{2}\eta_{i}\left(t\right)$$

(2) $$\tau_{\theta }\frac{d\theta }{dt}=-\theta +\frac{\text{Ω}}{N}\overset{N}{\underset{i=1}{\sum }}\varphi \left(x_{i},\gamma ,\theta \right)$$

(3) $$\varphi \left(x_{i},\gamma ,\theta \right)=\frac{1}{2}\left(1+\text{tanh}\left(\gamma \left(x_{i}-\theta \right)\right)\right)$$

where *W *is the N = 66 nodes human connectome [3], *x *is the node potential, *φ *is the node output, *θ *is the adaptive threshold, *η*(*t*) is a white noise and σ the diffusion parameter. Three main parameters (excitation strength ||*W*||, inhibition strength *Ω *and node excitability γ) are systematically varied in order to identify the fixed points attractors of the dynamics (Figure 1).

The adaptive threshold plays the role of a global inhibition and allows stabilizing many attractors, where the Ω / ||W|| ratio controls the sparsity of the activity. Then multistable dynamical systems are obtained in a large region of the parameter space, allowing to identify many different attractor patterns. The number of attractors is found to increase with the value of gamma (node excitability), at the expense of their intrinsic stability. Itinerant dynamics is obtained when a significant noise level is introduced in the system. Then, several "attractive" patterns can be reached on a single trajectory, where the duration of the visit reflects the stability of the pattern.

The simple dynamical system we have implemented allows exploring a large region of the parameters space, but is also capable of reproducing some aspects of the brain's spontaneous activity (switching between different attractors). The set of attractors we find in some regions of the parameters space share similarities with the different functional modes observed in the resting-state activity [1].
